# Predictors of complete miscarriage after expectant management or misoprostol treatment of non-viable early pregnancy in women with vaginal bleeding

**DOI:** 10.1007/s00404-020-05672-6

**Published:** 2020-07-07

**Authors:** Anna Fernlund, Ligita Jokubkiene, Povilas Sladkevicius, Lil Valentin

**Affiliations:** 1 Department of Obstetrics and Gynecology, Skåne University Hospital, Jan Waldenströms gata 47, 20502 Malmö, SE Sweden; 2 Department of Clinical Sciences Malmö, Lund University, Lund, Sweden

**Keywords:** First-trimester pregnancy, Misoprostol, Pregnancy complications, Logistic models prediction, Miscarriage

## Abstract

**Purpose:**

To identify predictors of complete miscarriage after expectant management or misoprostol treatment of non-viable early pregnancy in women with vaginal bleeding.

**Methods:**

This was a planned secondary analysis of data from a published randomized controlled trial comparing expectant management with vaginal single dose of 800 µg misoprostol treatment of women with embryonic or anembryonic miscarriage. Predefined variables—serum-progesterone, serum-β-human chorionic gonadotropin, parity, previous vaginal deliveries, gestational age, clinical symptoms (bleeding and pain), mean diameter and shape of the gestational sac, crown-rump-length, type of miscarriage, and presence of blood flow in the intervillous space—were tested as predictors of treatment success (no gestational sac in the uterine cavity and maximum anterior–posterior intracavitary diameter was ≤ 15 mm as measured with transvaginal ultrasound on a sagittal view) in univariable and multivariable logistic regression.

**Results:**

Variables from 174 women (83 expectant management versus 91 misoprostol) were analyzed for prediction of complete miscarriage at ≤ 17 days. In patients managed expectantly, the rate of complete miscarriage was 62.7% (32/51) in embryonic miscarriages versus 37.5% (12/32) in anembryonic miscarriages (*P* = 0.02). In multivariable logistic regression, the likelihood of success increased with increasing gestational age, increasing crown-rump-length and decreasing gestational sac diameter. Misoprostol treatment was successful in 80.0% (73/91). No variable predicted success of misoprostol treatment.

**Conclusions:**

Complete miscarriage after expectant management is significantly more likely in embryonic miscarriage than in anembryonic miscarriage. Gestational age, crown-rump-length, and gestational sac diameter are independent predictors of success of expectant management. Predictors of treatment success may help counselling women with early miscarriage.

## Introduction

Early miscarriage occurs in 10–15% of clinically recognized pregnancies [1–3]. Expectant or medical management are alternatives to surgical evacuation [4–6]. Randomized trials comparing medical treatment, most often misoprostol, with expectant management or placebo show substantial variation in success rates defined as complete miscarriage without surgical intervention [4–10]. The discrepancies are explained by differences in types of miscarriage included, symptomatology, definition of complete miscarriage and treatment success, and dose regimens of drugs. First-trimester miscarriages can be classified on ultrasound as (1) anembryonic, i.e., a gestational sac that is empty or with minimal embryonic debris without cardiac activity [11], (2) embryonic, i.e., a gestational sac with a visible embryo or fetus without cardiac pulsations [11], or as (3) incomplete miscarriage, i.e., no visible gestational sac but ultrasound signs of retained products of conception [12]. Incomplete miscarriages usually resolve spontaneously within a few weeks [12]. Expectant management of miscarriages with a retained gestational sac is less likely to be successful within a few weeks, especially in women with no vaginal bleeding [13]. Most studies investigating possible predictors of treatment success of medical treatment or expectant management include both incomplete miscarriages and embryonic or anembryonic miscarriages and/or both patients with and without vaginal bleeding [13–32]. This makes results difficult to generalize.

The aim of this work is to identify predictors of success of expectant management or misoprostol treatment in a well-defined group of patients, i.e., patients with embryonic or anembryonic miscarriage reporting vaginal bleeding.

## Material and methods

This is a planned secondary analysis of prospectively collected data in a published randomized controlled trial comparing expectant management with vaginal single dose 800 µg misoprostol treatment of embryonic and anembryonic first-trimester miscarriage [6] (ClinicalTrials.gov ID: NCT01033903). The regional ethical review board, Lund University, Sweden, approved the trial (Dnr 83/2008), which was carried out in accordance with the code of ethics of the Declaration of Helsinki. The primary trial outcome was complete miscarriage ≤ 10 weeks. Secondary outcomes were complete miscarriage ≤ 17, ≤ 24 and ≤ 31 days. Complete miscarriage was defined as no gestational sac in the uterine cavity and maximum anterior–posterior diameter of the intracavitary contents ≤ 15 mm as measured with transvaginal ultrasound on a sagittal view. Trial details (including a CONSORT flow chart) have been published [6] and are briefly outlined below.

Women consulting the gynecological emergency clinic of Skåne University Hospital, Malmö, Sweden and reporting vaginal bleeding in early pregnancy were eligible for inclusion if hemodynamically stable. Women with heavy bleeding needing urgent surgical evacuation of the uterine cavity, as judged clinically, were not eligible. Inclusion criteria were: ≥ 18 years old, understanding written and spoken Swedish, hemoglobin concentration > 80 g/L, no contraindications to misoprostol treatment, fulfilling ultrasound criteria of anembryonic or embryonic miscarriage [11], and fetal crown-rump-length ≤ 33 mm. Because of new recommendations [33–35] our ultrasound criteria of non-viability were changed in 2014. Before 2014 the criteria were (1) intracavitary gestational sac with a diameter (mean of three orthogonal diameters) of > 16 mm with no embryonic pole [36] or (2) intracavitary gestational sac with an embryo with crown-rump-length ≥ 5 mm without cardiac pulsations [36], or (3) if the above criteria were not fulfilled, intracavitary gestational sac with or without an embryo showing no significant development at a repeat scan after 7 days [36]. After April 2014 our non-viability criteria were: (1) intracavitary gestational sac with a mean diameter ≥ 25 mm with no embryonic pole, or (2) intracavitary gestational sac with an embryo with crown-rump-length ≥ 7 mm without cardiac pulsations, or (3) if the above criteria were not fulfilled, no significant development at a repeat scan after 7 days [33, 34, 37, 38]. Women with incomplete miscarriage were not eligible. Women giving written consent were randomized into two parallel groups in an open-label 1:1 ratio to misoprostol treatment or expectant management. All patients were managed as outpatients.

The patients were examined clinically and with transvaginal ultrasound in the lithotomy position on the day of randomization. The trial clinician estimated bleeding and pain at speculum and clinical examination and placed 800 μg misoprostol in the posterior vaginal fornix of the patients allocated to medical treatment. The first follow-up was after 10 days. Subsequent follow-up visits were scheduled every 7 days until the miscarriage was complete (definition, see above). The patient was then discharged with no planned follow-up visits. If complete evacuation was not achieved on day 31, dilatation and evacuation was recommended. However, the participants could ask for dilatation and evacuation at any time for any reason during the trial.

At inclusion, information on demographic background data was collected from the woman and documented in research forms, and blood was drawn for analysis of hemoglobin, β-human chorionic gonadotropin (β-hCG), progesterone and blood type. hCG + β was measured with a sandwich immunoassay on a Cobas® instrument; Intact hCG + the β-subunit assay (Roche Diagnostics, Mannheim, Germany). Progesterone was measured with a competitive immunoassay on a Cobas® instrument; Progesterone III assay (Roche Diagnostics, Mannheim, Germany). On every follow-up visit clinical examination and transvaginal ultrasound were performed. Results were prospectively entered into research forms.

The ultrasound machine used was a Sequoia 512 ultrasound system (Siemens Medical Solutions Inc., Ultrasound Division, Mountain View, CA, USA) with a 4–8 MHz transducer. The shape of the gestational sac was assessed, the sac diameter and the crown-rump-length (if an embryo/fetus was present) were measured. The miscarriage was classified as embryonic or anembryonic. Assessment of blood flow in the presumed intervillous space was first made by looking for flickering areas within the chorion on grey-scale imaging. The color Doppler function was then switched on starting with standardized settings (space–time S2; edge zero; persistence two; color map V2; gate two; filter three; frequency 7 MHz; color Doppler gain 50; pulse repetition frequency corresponding to blood flow velocity 2.1 cm/s) which were adjusted to maximize detection of slow velocity blood flow without artifacts. A Doppler gate was placed where color Doppler signals were seen inside the chorion. By adjusting the position of the probe, arterial Doppler shift signals inside the chorion were searched for as previously described [39, 40]. Presence of blood flow in the presumed intervillous space as assessed on grey scale and color Doppler and of arterial Doppler shift signals in the presumed intervillous space was noted.

Women not showing up on a scheduled visit were included in this secondary analysis only if on a later visit the miscarriage was incomplete; the miscarriage was then classified as incomplete also on the missed visit. Women with a complete miscarriage on the first visit after the missed one were not included in the analysis, because we do not know, if the miscarriage was complete also on the missed visit.

We considered the following variables to be possibly related to treatment outcome and explored their ability to predict complete miscarriage ≤ 10 days and ≤ 17 days: serum/plasma levels of progesterone (nmol/L) and β-hCG (IU/L) at inclusion, gestational age (days) according to the last menstrual period (LMP), previous vaginal delivery (yes/no), parity (yes/no), bleeding at inclusion (moderate or heavy versus mild or none as assessed by the trial physician at speculum examination), pain at inclusion (yes/no), shape of the gestational sac (round or oval versus else), mean gestational sac diameter (mm), crown-rump-length (mm), type of miscarriage (embryonic or anembryonic), presence of blood flow in the presumed intervillous space according to grey scale and color Doppler ultrasound (yes/no), and presence of arterial Doppler shift signals in the presumed intervillous space (yes/no).

Statistical analysis was performed using SPSS Statistics, version 21 (IBM Corp., Armonk, NY, USA). It was done separately for the misoprostol group and the expectantly managed group and separately for treatment success ≤ 10 days and ≤ 17 days. The relation between the predefined predictor variables and treatment success was tested for statistical significance using univariable logistic regression with the likelihood ratio test. Two-tailed *P* values < 0.05 were considered statistically significant. Multivariable logistic regression was used to elucidate which variables were independently associated with treatment success and for building models to predict complete miscarriage. Because crown-rump-length and miscarriage type are related (in anembryonic miscarriages the crown-rump-length is zero), only one of these variables was included in the same multivariable analysis. We used different approaches for model building. In one, we included all predetermined variables (with a minimum of five individuals in each cell of a four-field table) in multivariable backward step-wise logistic regression analysis. In another, we started with including only variables with a *P* value < 0.20 in univariable analysis, and then we tested to add variables that we found clinically relevant.

Individual data for each patient were inserted into the regression models to calculate the probability of complete miscarriage for each patient and to plot receiver-operating characteristics curves. The area under the receiver-operating characteristic curve (AUC) and its 95% confidence interval (CI) were calculated. If the lower limit of the CI was > 0.5 the model was considered to have discriminatory ability. The larger the AUC the better the discriminative ability.

## Results

Between September 2008 and December 2015 189 women were recruited into the trial. Ninety-five women were allocated to expectant management and 94 to misoprostol treatment. Twenty-one women (expectant group: 11; misoprostol group: 10) were recruited after the new ultrasound criteria of non-viability were adopted. After exclusions, our planned secondary analysis included 177 women (expectant group: 85; misoprostol group: 92). The reasons for exclusion were: withdrawal of consent (expectant group, *n* = 2), not fulfilling inclusion criteria (expectant group, *n* = 3), undergoing dilatation and evacuation before first follow-up at 10 days (expectant group, *n* = 5; misoprostol group, *n* = 2). For the analysis regarding prediction of complete miscarriage ≤ 17 days another three patients were excluded due to dilatation and evacuation on patient’s request (expectant group *n* = 1) or missed follow-up visit (expectant group *n* = 1, misoprostol group *n* = 1), the numbers analyzed for this outcome being 83 (expectant group) and 91 (misoprostol group).

Expectant management was successful ≤ 10 days in 45.8% (39/85) of the patients and ≤ 17 days in 53.0% (44/83). Variables associated with success of expectant management in univariable and multivariable analyses are shown in Tables 1, 2, 3, 4 and 5. Treatment success was more common in embryonic than anembryonic miscarriages: complete miscarriage ≤ 10 days 53.8% (28/52) versus 33.0% (11/33) (*P* = 0.06), complete miscarriage ≤ 17 days 62.7% (32/51) versus 37.5% (12/32) (*P* = 0.02). In multivariable analyses, the following variables were independently associated with treatment success: gestational age according to LMP (the higher the more likely successful treatment), mean gestational sac diameter (the smaller the more likely successful treatment) and crown-rump-length (the larger the more likely successful treatment). When we replaced crown-rump-length with type of miscarriage in the multivariable model, the odds of treatment success were approximately six times higher in embryonic than anembryonic miscarriages (Table 3). When s-β-hCG and s-progesterone were tested as predictors in multivariable analysis, either s-progesterone or s-β-hCG (not both) and crown-rump-length or miscarriage type were independently associated with complete miscarriage (Tables 4 and 5). The AUCs of the models ranged from 0.71 to 0.77.

**Table 1:** Association between predefined possible predictors and success of expectant management of embryonic and anembryonic miscarriage ≤ 10 days (univariable logistic regression analysis); *n* = 85

Variables tested as possible predictors	Success (*n* = 39)	Failure (*n* = 46)	*P* value^a^	Odds ratio (95% CI)
Biochemical variables				
s-beta-human chorionic gonadotropin (IU/L)^b^		2 missing	0.01	0.956 (0.919–0.996)^c^
Mean	9387.6 ± 7690.1	17,744.8 ± 19,865.8		
Median	7650.0 (107–26,585)	9328.5 (1006–80,317)		
s-Progesterone (nmol/L)^b^		1 missing	0.011	0.971 (0.942–1.000)
Mean	18.9	32.3		
Median	18.0 (3–41)	19.0 (4–190)		
Clinical variables				
Gestational age according to last menstrual period	2 missing	3 missing	0.053	1.040 (0.998–1.083)
Mean	77.8 ± 11.1	72.9 ± 11.7		
Median	76.0 (60–104)	73.0 (42–94)		
Vaginal delivery				
Yes	20 (51.3)	28 (60.9)	0.374	0.677 (0.285–1.604)
No	19 (48.7)	18 (39.1)		
Parity				
Parous	24 (61.3)	29 (63.0)	0.887	0.938 (0.389–2.262)
Nulliparous	15 (38.5)	17 (37.0)		
Bleeding at inclusion^d^				
Moderate/heavy	8 (20.5)	5 (10.9)	0.218	2.116 (0.631–7.102)
None/mild	31 (79.5)	41 (89.1)		
Pain at inclusion				
Yes	12 (30.8)	12 (26.1)	0.633	1.259 (0.489–3.244)
No	27 (69.2)	34 (73.9)		
Ultrasound variables				
Gestational sac			0.624	0.788 (0.303–2.047)
Not round/oval	10 (25.6)	14 (30.4)		
Round/oval	29 (74.4)	32 (69.6)		
Gestational sac diameter (mm)^e^		1 missing	0.448	0.981 (0.933–1.031)
Mean	23.3	24.5		
Median	21.0 (4.7–49.0)	23.3 (10.7–41.0)		
Crown-rump-length (mm)			0.008	1.102 (1.020–1.191)
Mean	7.5 ± 7.3	4.0 ± 4.9		
Median	5.5 (0.0–26.0)	3.1 (0.0–17.0)		
Miscarriage type on ultrasound				
Embryonic	28 (71.8)	24 (52.2)	0.063	2.333 (0.943–5.774)
Anembryonic	11 (28.2)	22 (47.8)		
Crown-rump-length if embryonic miscarriage (mm)	*n* = 28	*n* = 24	0.06	1.105 (0.988–1.235)
Mean	10.5 ± 6.6	7.6 ± 4.3		
Median	9.5 (3.0–26.0)	5.5 (3.0–17.0)		
Blood flow in presumed intervillous space according to				
Grey-scale ultrasound			0.288	0.398 (0.069–2.299)
Yes	35 (89.7)	44 (95.7)		
No	4 (10.4)	2 (4.3)		
Color Doppler ultrasound		1 missing	0.109	0.327 (0.078–1.363)
Yes	32 (82.1)	42 (93.3)		
No	7 (17.9)	3 (6.7)		
Spectral Doppler ultrasound (arterial blood flow)	4 missing	5 missing	0.619	0.786 (0.304–2.032)
Yes	22 (62.9)	27 (65.9)		
No	13 (37.1)	14 (34.1)		

Data are presented as *n* (%), mean ± SD or median (range)

*CI* confidence interval

^a^Likelihood ratio test

^b^After mid May 2013 beta-human chorionic gonadotropin and progesterone were analyzed in plasma not in serum, reference intervals are not affected

^c^Odds ratio is calculated for units of 1000

^d^Bleeding as judged by the trial physician at speculum examination—all women reported vaginal bleeding

^e^Mean of three orthogonal diameters

**Table 2:** Association between predefined possible predictors and success of expectant management of embryonic and anembryonic miscarriage ≤ 17 days (univariable logistic regression analysis); *n* = 83

Variables tested as possible predictors	Success (*n* = 44)	Failure (*n* = 39)	*P* value^a^	Odds ratio (95% CI)
Biochemical variables				
s-beta-human chorionic gonadotropin (IU/L)^b^		2 missing	0.01	0.958 (0.922–0.995)^c^
Mean	9530.6 ± 7483.3	18,128.3 ± 21,019.9		
Median	8308.0 (107–26,585)	7990.0 (1006–80,317)		
s-Progesterone (nmol/L)^b^		1 missing	0.003	0.965 (0.936–0.995)
Mean	18.5 ± 10.6	34.6 ± 38.2		
Median	18.0 (3–41)	20.5 (4–190)		
Clinical variables				
Gestational age according to last menstrual period (days)	2 missing	3 missing	0.04	1.042 (1.000–1.086)
Mean	77.8 ± 10.7	72.4 ± 12.4		
Median	78.0 (60–104)	72.0 (42–94)		
Vaginal delivery				
Yes	25 (56.8)	23 (59.0)	0.843	0.915 (0.382–2.195)
No	19 (43.2)	16 (41.0)		
Parity				
Yes	29 (65.9)	24 (61.5)	0.679	1.208 (0.493–2.963)
No	15 (34.1)	15 (38.5)		
Bleeding at inclusion^d^				
Moderate/heavy	10 (22.7)	3 (7.7)	0.053	3.529 (0.894–13.927)^e^
None/mild	34 (77.3)	36 (92.3)		
Pain at inclusion				
Yes	13 (29.5)	11 (28.2)	0.893	1.067 (0.412–2.765)
No	31 (70.5)	28 (71.8)		
Ultrasound variables				
Gestational sac			0.925	0.955 (0.365–2.499)
Not round/oval	12 (27.3)	11 (28.2)		
Round/oval	32 (72.7)	28 (71.8)		
Gestational sac diameter (mm)^f^		1 missing	0.967	0.999 (0.950–1.050)
Mean	24.1 ± 9.5	24.1 ± 7.9		
Median	21.4 (4.7–49.0)	23.2 (10.7–41.0)		
Crown-rump-length (mm)				
Mean	7.5 ± 7.0	3.6 ± 4.9	0.004	1.119 (1.028–1.218)
Median	5.6 (0.0–26.0)	0.0 (0.0–17.0)		
Miscarriage type on ultrasound				
Embryonic	32 (72.7)	19 (48.7)	0.024	2.807 (1.126–6.998)
Anembryonic	12 (27.3)	20 (51.3)		
Crown-rump-length if embryonic miscarriage (mm)	*n* = 32	*n* = 19	0.069	1.109 (0.981–1.254)
Mean	10.3 ± 6.2	7.4 ± 4.6		
Median	9.5 (3.0–26.0)	5.3 (3.0–17.0)		
Blood flow in presumed intervillous space according to				
Grey-scale ultrasound			0.105	0.205 (0.023–1.840)
Yes	39 (88.6)	38 (97.4)		
No	5 (11.4)	1 (2.6)		
Color Doppler ultrasound		1 missing	0.008	0.105 (0.013–0.873)
Yes	35 (79.5)	37 (94.9)		
No	9 (20.5)	1 (2.6)		
Spectral Doppler ultrasound (arterial blood flow)	4 missing	4 missing	0.125	0.468 (0.175–1.252)
Yes	23 (57.5)	25 (71.4)		
No	17 (42.5)	10 (28.6)		

Data are presented as *n* (%), mean ± SD, median (range)

*CI* confidence interval

^a^Likelihood ratio test

^b^After mid May 2013 beta-human chorionic gonadotropin and progesterone were analyzed in plasma and not in serum, reference intervals are not affected

^c^Odds ratio is calculated for units of 1000

^d^Bleeding as judged by the trial physician at speculum examination—all women reported vaginal bleeding

^e^We consider this result unreliable, because only three patients in the failure group had heavy or moderate bleeding, the unreliable result is also reflected in the odds ratio

^f^Mean of three orthogonal diameters

**Table 3:** Results of multivariable logistic regression analysis showing variables independently associated with complete miscarriage ≤ 10 and ≤ 17 days in women with embryonic or anembryonic miscarriage managed expectantly

	Model with crown-rump-length					Model with miscarriage type				
	AUC (95% CI)	Constant	Coefficient	Odds ratio (95% CI)	*P* value^a^	AUC	Constant	Coefficient	Odds ratio (95% CI)	*P* value^a^
Complete miscarriage ≤ 10 days (*n* = 79)										
	0.764	− 3.691				0.739	− 5.058			
	(0.659–0.869)					(0.628–0.850)				
Variables in model										
Gestational age according to last menstrual period (OR for change in days)			0.08	1.083 (1.023–1.146)	0.003			0.083	1.087 (1.024–1.153)	0.002
Mean sac diameter (OR for change in mm)			− 0.146	0.864 (0.788–0.948)	0.001			− 0.099	0.905 (0.838–0.978)	0.007
CRL (OR for change in mm)			0.202	1.224 (1.086–1.380)	0			NA	NA	NA
Type of miscarriage (embryonic or anembryonic)			NA	NA	NA			1.737	5.682 (1.724–18.730)	0.002
Complete miscarriage ≤ 17 days (*n* = 79)										
	0.763	− 3.382				0.754	− 4.783			
	(0.658–0.869)					(0.645–0.863)				
Variables in model										
Gestational age according to last menstrual period (OR for change in days)			0.07	1.072 (1.015–1.133)	0.008			0.076	1.079 (1.017–1.144)	0.006
Mean sac diameter (OR for change in mm)			− 0.111	0.895 (0.818–0.979)	0.01			− 0.076	0.927 (0.858–1.001)	0.046
CRL (OR for change in mm)			0.194	1.214 (1.074–1.372)	0			NA	NA	NA
Type of miscarriage (embryonic or anembryonic)			NA	NA	NA			1.802	6.060 (1.837–19.992)	0.001

The probability of treatment success is calculated as [*e*^*z*^/(1 + *e*^*z*^) where *e* = 2.718 (base value of natural logarithm) and *z* is calculated as:

*z* = constant + (coefficient × gestational age according to last menstrual period in days + coefficient × crown-rump length in mm + coefficient × mean sac diameter in mm)

or

*z* = constant + (coefficient × gestational age according to last menstrual period in days + coefficient × type of miscarriage where anembryonic gestation is coded as 0 and fetal demise as 1 + coefficient × mean sac diameter in mm)

*AUC* area under receiver-operating characteristic curve, *CI* confidence interval, *OR* odds ratio, *CRL* crown-rump-length, *NA* not applicable

^a^Likelihood ratio test

**Table 4:** Results of multivariable logistic regression analysis showing variables independently associated with complete miscarriage ≤ 10 and ≤ 17 days in women with embryonic or anembryonic miscarriage managed expectantly when s-β-hCG was included as a variable

	Model with crown-rump-length					Model with miscarriage type				
	AUC (95% CI)	Constant	Coefficient	Odds ratio (95% CI)	*P* value^a^	AUC (95% CI)	Constant	Coefficient	Odds ratio (95% CI)	*P* value^a^
Complete miscarriage ≤ 10 days (*n* = 83)										
	0.741	− 0.212				0.714	− 0.177			
	(0.634–0.847)					(0.603–0.825)				
Variables in model										
s-β-hCG (OR for change in units of 1000 IU/L)			− 0.044	0.957 (0.919–0.997)	0.013			− 0.052	0.949 (0.910–0.990)	0.004
CRL (OR for change in mm)			0.13	1.139 (1.036–1.252)	0.002			NA	NA	NA
Type of miscarriage (embryonic or anembryonic)			NA	NA	NA			1.177	3.243 (1.227–8.571)	0.015
Complete miscarriage ≤ 17 days (*n* = 81)										
	0.768	− 0.024				0.735	0.008			
	(0.664–0.872)					(0.623–0.846)				
Variables in model										
s-β-hCG (OR for change in units of 1000 IU/L)			− 0.045	0.956 (0.918–0.995)	0.01			− 0.052	0.949 (0.910–0.989)	0.003
CRL (OR for change in mm)			0.169	1.185 (1.057–1.327)	0			NA	NA	NA
Type of miscarriage (embryonic or anembryonic)			NA	NA	NA			1.405	4.077 (1.505–11.046)	0.004

The probability of treatment success is calculated as [*e*^*z*^/(1 + *e*^*z*^) where *e* = 2.718 (base value of natural logarithm) and *z* is calculated as:

*z* = constant + (coefficient × s-beta-hCG in units of 1000 IU/L + coefficient × crown-rump length in mm)

or

*z* = constant + (coefficient × s-beta-hCG in units of 1000 IU/L + coefficient × type of miscarriage where anembryonic gestation is coded as 0 and fetal demise as 1)

*s-β-hCG* serum β-human chorionic gonadotropin, *AUC* area under receiver-operating characteristic curve, *CI* confidence interval, *CRL* crown-rump-length, *OR* odds ratio, *NA* not applicable

^a^Likelihood ratio test

**Table 5:** Results of multivariable logistic regression analysis showing variables independently associated with complete miscarriage ≤ 10 days and ≤ 17 days in women with embryonic or anembryonic miscarriage managed expectantly when s-progesterone was included as a variable

	Model with crown-rump-length					Model with miscarriage type				
	AUC (95% CI)	Constant	Coefficient	Odds ratio (95% CI)	*P* value^a^	AUC (95% CI)	Constant	Coefficient	Odds ratio (95% CI)	*P* value^a^
Complete miscarriage ≤ 10 days (*n* = 84)										
	0.716	− 0.077				0.711	− 0.032			
	(0.607–0.826)					(0.601–0.821)				
Variables in model										
s-Progesterone (OR for change in nmol/L)			− 0.03	0.971 (0.945–0.997)	0.006			− 0.034	0.967 (0.938–0.996)	0.004
CRL (OR for change in mm)			0.126	1.135 (1.035–1.244)	0.002			NA	NA	NA
Type of miscarriage (embryonic or anembryonic)			NA	NA	NA			1.122	3.070 (1.182–7.978)	0.018
Complete miscarriage ≤ 17 days (*n* = 82)										
	0.765	0.263				0.749	0.284			
	(0.662–0.867)					(0.642–0.857)				
Variables in model										
s-Progesterone (OR for change in nmol/L)			− 0.037	0.964 (0.937–0.991)	0.001			− 0.041	0.960 (0.930–0.991)	0.001
CRL (OR for change in mm)			0.165	1.180 (1.056–1.318)	0.001			NA	NA	NA
Type of miscarriage (embryonic or anembryonic)			NA	NA	NA			1.408	4.086 (1.513–11.034)	0.004

The probability of treatment success is calculated as [*e*^*z*^/(1 + *e*^*z*^) where *e* = 2.718 (base value of natural logarithm) and *z* is calculated as:

*z* = constant + (coefficient × s-Progesterone in nmol/L + coefficient × crown-rump length in mm)

or

*z* = constant + (coefficient × s-Progesterone in nmol/L + coefficient × type of miscarriage where anembryonic gestation is coded as 0 and fetal demise as 1)

*s-progesterone* serum-progesterone, *AUC* area under receiver-operating characteristic curve, *CI* confidence interval, *OR* odds ratio, *CRL* crown-rump-length, *NA* not applicable

^a^Likelihood ratio test

In embryonic miscarriages, the higher the gestational age according to LMP, the larger the crown-rump-length and the smaller the mean gestational sac diameter the higher the likelihood of complete miscarriage ≤ 10 and ≤ 17 days (Table 6); and the lower the s-progesterone or s-β-hCG and the longer the crown-rump-length the higher the success rate (Table 7). The AUCs of the models ranged from 0.80 to 0.84. No variable was statistically significantly associated with complete miscarriage of anembryonic miscarriages when using expectant management (details available from the authors on request).

**Table 6:** Results of multivariable logistic regression analysis showing variables independently associated with complete miscarriage ≤ 10 days and ≤ 17 days in women with embryonic miscarriage managed expectantly

	AUC (95% CI)	Constant	Coefficient	Odds ratio (95%)	*P* value
Complete miscarriage ≤ 10 days (*n* = 48)					
	0.843	− 5.397			
	(0.732–0.954)				
Variables in model					
Gestational age according to last menstrual period (OR for change in days)			0.131	1.140 (1.024–1.268)	0.005
Mean sac diameter (OR for change in mm)			− 0.226	0.797 (0.693–0.918)	0
CRL (OR for change in mm)			0.222	1.249 (1.036–1.505)	0.007
Complete miscarriage ≤ 17 days (*n* = 47)					
	0.802	− 5.609			
	(0.657–0.948)				
Variables in model					
Gestational age according to last menstrual period (OR for change in days)			0.129	1.137 (1.022–1.266)	0.006
Mean sac diameter (OR for change in mm)			− 0.177	0.838 (0.733–0.958)	0.002
CRL (OR for change in mm)			0.17	1.186 (0.987–1.424)	0.043

The probability of treatment success is calculated as [*e*^*z*^/(1 + *e*^*z*^) where *e* = 2.718 (base value of natural logarithm) and *z* is calculated as:

*z* = constant + (coefficient × gestational age according to last menstrual period in days + coefficient × mean sac diameter in mm + coefficient × crown-rump length in mm)

*AUC* area under receiver-operating characteristic curve, *CI* confidence interval, *OR* odds ratio, *CRL* crown-rump-length

^a^Likelihood ratio test

**Table 7:** Results of multivariable logistic regression analysis showing variables independently associated with complete miscarriage ≤ 10 days and ≤ 17 days in women with embryonic miscarriage managed expectantly when s-β-hCG or s-progesterone were included as variables

	Model with s-hCG						Model with s-progesterone				
	AUC (95% CI)	Constant	Coefficient	Odds ratio (95% CI)	*P* value^a^		AUC (95% CI)	Constant	Coefficient	Odds ratio (95% CI)	*P* value^a^
Complete miscarriage ≤ 10 days (*n* = 49)						(*n* = 50)					
	0.821						0.834				
	(0.707–0.936)	2.857					(0.726–0.943)	− 0.032			
Variables in model											
s-β-hCG (OR for change in units of 1000 IU/L)			− 0.066	0.936 (0.866–1.011)	0.035				NA	NA	NA
s-Progesterone (OR for change in nmol/L)			NA	NA	NA				− 0.038	0.963 (0.920–1.008)	0.006
Mean sac diameter (OR for change in mm)			− 0.145	0.865 (0.775–0.966)	0.004				− 0.159	0.853 (0.761–0.957)	0.002
CRL (OR for change in mm)			0.254	1.289 (1.052–1.581)	0.003				0.289	1.335 (1.074–1.659)	0.001
Complete miscarriage ≤ 17 days (*n* = 49)						(*n* = 49)					
	0.796						0.824				
	(0.654–0.938**)**	0.348					(0.704–0.943)	3.072			
Variables in model											
s-β-hCG (OR for change in units of 1000 IU/L)			− 0.061	0.941 (0.889–0.996)	0.008				NA	NA	NA
s-Progesterone (OR for change in nmol/L)			NA	NA	NA				− 0.043	0.958 (0.918–1.000)	0.001
Mean sac diameter (OR for change in mm)			NA	NA	NA				− 0.114	0.893 (0.797–1.000)	0.036
CRL (OR for change in mm)			0.152	1.165 (0.973–1.395)	0.049				0.27	1.311 (1.037–1.656)	0.006

The probability of treatment success is calculated as [*e*^*z*^/(1 + *e*^*z*^) where *e* = 2.718 (base value of natural logarithm) and *z* is calculated as:

*z* = constant + (coefficient × s-beta-hCG in units of 1000 IU/L + coefficient × mean sac diameter in mm + coefficient × crown-rump length in mm)

or

*z* = constant + (coefficient × s-progesterone in nmol/L + coefficient × mean sac diameter in mm + coefficient × crown-rump length in mm)

*s-β-hCG* serum beta-human chorionic gonadotropin, *s-progesterone* serum-progesterone, *AUC* area under receiver-operating characteristic curve, *CI* confidence interval, *OR* odds ratio, *CRL* crown-rump length, *NA* not applicable

^a^Likelihood ratio test

Misoprostol treatment was successful ≤ 10 days in 67% (62/92) of patients and ≤ 17 days in 80% (73/91). No variable predicted success of misoprostol treatment either in embryonic or anembryonic miscarriages. (Details available from the authors on request).

## Comments

In patients managed expectantly the likelihood of spontaneous complete miscarriage ≤ 10 or ≤ 17 days was twice as high in embryonic as in anembryonic miscarriages. The likelihood of complete miscarriage increased with increasing gestational age according to LMP, increasing crown-rump-length and decreasing gestational sac diameter; and the larger the crown-rump-length and the lower the s-β-hCG or s-progesterone level the higher the likelihood of treatment success. No variable predicted treatment outcome in the misoprostol group.

The strength of our study is the well-defined study population. We included only embryonic and anembryonic miscarriages and only women reporting vaginal bleeding. Our results are generalizable to such women. It is a limitation that we changed our definitions of non-viable pregnancy at the end of the recruitment period because of new research results and guidelines [33–35, 37, 38, 41–43]. However, it is unlikely that this had any major impact on our results. Our definition of successful treatment, also used by others [7, 8, 19, 27, 44], may also be criticized [45–48]. Absence of a gestational sac and/or cessation of vaginal bleeding may be better definitions [49]. It is a weakness that our prediction models have not been validated in a new study sample. In our trial, we did not find any association between presence of blood flow or pulsatile flow in the intervillous space and treatment success. However, we did not make any attempts to quantify blood flow, for example using three-dimensional ultrasound and calculating vascular indices. This may be seen as a limitation.

The results of other studies exploring possible predictors of complete miscarriage with expectant management are very heterogenous (Table 8) [13–22, 25, 30]. This is probably explained by differences in study populations (types of miscarriage, symptomatology), definitions of complete miscarriage and treatment success, and variables tested as predictors. However, more than one study reported that the lower the s-β-hCG and s-progesterone values the higher the likelihood of success of expectant management [16, 18, 19, 21] and that success rate is higher in incomplete miscarriages than in embryonic or anembryonic miscarriages [15–17]. Only two studies are reasonably similar to ours with regard to inclusion criteria and definition of treatment success [18, 21]. Schwärzler et al. found that pulsatile flow in the presumed intervillous space was a predictor of successful treatment [21]. We could not confirm this, perhaps because examination of blood flow in the intervillous space in early pregnancy requires skill, carefulness and time. Therefore, it is unlikely to be useful in busy emergency departments or early pregnancy units. Memtsa et al. reported that the older the patient and the lower the s-progesterone level the higher the likelihood of complete miscarriage < 7 days [18]. We did not test patient age as a predictor, because we found it unlikely to be related to the time to complete evacuation of the uterine cavity.

**Table 8:** Published studies exploring possible predictors of outcome of expectant management or medical treatment of early miscarriage

First author, year	Rate of success	Inclusion criteria (type of miscarriage, symptoms)	Definition of successful treatment	Variables tested as predictors	Variables predictive of success in univariable analysis^b^	Variables predictive of success in multivariable analysis^b^
Study type						
Number of patients						
Expectant management						
Acharya 2002 [14]	54% (46/86)	Embryonic (59%), anembryonic (41%); bleeding or not bleeding is not stated	Complete miscarriage within 4 weeks (no heavy bleeding and AP diameter of uterine contents < 15 mm)	Maternal age, menstrual age, parity, abortion, CRL, mean sac diameter, gestational sac volume	Menstrual age (+)	Not performed
Prospective observational						
* N* = 86						
Jurkovic 1998 [13]	25% (21/85)	Embryonic, anembryonic; both bleeding and no bleeding	Absence of vaginal bleeding and no evidence of RPOC on TVS (no cut off in mm); no time limit	Not clearly stated: Maternal age, parity, number of previous miscarriages, menstrual age, vaginal bleeding, mean gestational sac diameter	Mean gestational sac diameter? (−), menstrual age (−)	Not performed
Prospective observational						
* N* = 85						
Elson 2005 [16]	69% (37/54)	Missed miscarriages (embryonic or anembryonic—41%); incomplete miscarriages (no gestational sac—59%); bleeding and mild lower abdominal pain	Complete miscarriage, i.e., bleeding settled and negative urine pregnancy test without D&E, no time limit; median time to resolution 7 days	s-β-hCG, s-progesterone, 17-hydroxy-progesterone, inhibin A, inhibin pro α-C RI, insulin growth factor-binding protein-1 (all in serum) Maternal age, menstrual age, bleeding yes or no, mean diameter of uterine contents, type of miscarriage	(Decision tree analysis) Diameter of RPOC (−), s-β-hCG (−), s-progesterone (−), inhibin A (−), inhibin pro α-C RI (−), type of miscarriage, i.e., incomplete (+) and non-viable pregnancy (−)	Not performed
Observational						
* N* = 54						
Luise 2002 [30]	2 weeks: 83% (184/221); 4 weeks: 91% (201/221)	Incomplete miscarriage < 13 weeks (heterogeneous irregular tissue with or without gestational sac not compatible with missed miscarriage or anembryonic pregnancy); probably all patients had bleeding	Complete miscarriage without D&E within 4 weeks (< 15 mm endometrial thickness and no bleeding or pain)	Presence of gestational sac (yes or no), endometrial thickness	No variable predicted the outcome of expectant management. Trend towards a decrease in proportion of success with increasing endometrial thickness	Not performed
Prospective observational						
* N* = 221						
Luise 2002 [17]	81% (367/451)	First-trimester miscarriage: Incomplete miscarriage (heterogeneous tissue with or without gestational sac with no cut off in mm), embryonic and anembryonic; probably all patients had bleeding	Complete miscarriage (endometrial thickness < 15 mm and absence of bleeding) ≤ 4 weeks	Type of miscarriage	*P* values not presented, Type of miscarriage: Incomplete (+ +), fetal demise (+), anembryonic pregnancy (−)	Not performed
Prospective observational						
* N* = 451						
Casikar 2013 [22]	77% (161/210)	Incomplete, embryonic and anembryonic; both symptomatic and asymptomatic (bleeding and/or pain)	Cessation of vaginal bleeding and the absence of RPOC (not defined) on TVS ≤ 2 weeks	Bleeding (“none”, “without clots”, “with clots”), pain (yes/no)	Presence of bleeding was significantly predictive of success in incomplete miscarriage. Pain was not predictive	Not performed
Prospective observational						
*N* = 210						
Casikar 2012 [25]	85% (134/158)	Incomplete (no gestational sac, hyperechoic material in the uterine cavity, no cut off in mm); both bleeding and no bleeding	Complete miscarriage within 2 weeks i.e., cessation of bleeding and the absence of RPOC (not defined, no cut off) on TVS	Maternal age, parity, number of vaginal deliveries, number of cesarean sections, previous miscarriages, previous termination of pregnancies, menstrual age, bleeding (yes or no), endometrial thickness, RPOC volume, power Doppler color score, color Doppler signals detectable (yes or no)	Vaginal bleeding (+), absence of color Doppler signals in RPOC (+)	Not performed
Prospective observational						
*N* = 158						
Nielsen 1996 [19]	79% (81/103)	Inevitable (not defined) or incomplete miscarriage < 13 weeks with intrauterine tissue and blood clots; AP diameter 15–50 mm on TVS; clinical signs of miscarriage (bleeding)	Complete miscarriage (AP diameter of uterine contents ≤ 15 mm) on TVS ≤ 3 days	α-fetoprotein, 17β-estradiol, CA125, 17α-hydroxyprogesterone, s-β-hCG, s-progesterone, daily change in s-β-hCG (all in serum) Patient age, menstrual age, parity, previous miscarriages, previous medical terminations, duration of bleeding, volume of intrauterine contents, diameter of intrauterine contents	Volume of intrauterine contents (−), diameter of intrauterine contents (−), 17β-estradiol (−), CA125 (+), 17α-hydroxyprogesterone (−), s-β-hCG (−), s-progesterone (−), daily change in s-β-hCG (+)	s-progesterone (−) daily hCG-change (+) CA125 (+) α-fetoprotein (−) intrauterine contents diameter (−) A model with only s-β-hCG, s-progesterone and intrauterine diameter was also tested (*P* values not presented)
Retrospective (randomized in earlier study)						
*N* = 103						
Schwartzler 1999 [21]	1 week: 54% (46/85); 4 weeks: 84% (71/85)	Embryonic and anembryonic pregnancies; vaginal bleeding and/or pain	Endometrial cavity thickness < 10 mm and negative urinary pregnancy test within 7 days	Age, parity, previous miscarriage, menstrual age, gestational sac diameter, diameter of intrauterine contents, s-β-hCG, s-progesterone, Hb, PI, RI, PSV in uterine arteries and spiral arteries, presence of blood flow in presumed intervillous space (PI, RI, PSV in intervillous space)	Presence of blood flow in the intervillous space was more common in success group, p value not reported	s-progesterone (−) and s-β-hCG (−); Doppler results were not included in multivariable analysis
Prospective observational						
*N* = 85						
Wieringa-de Waard 2003 [20]	51% (95/188)	Early miscarriage < 16 weeks (embryonic and anembryonic) and incomplete miscarriage (uterine contents > 15 mm AP diameter on TVS); both bleeding and no bleeding	Uterine contents < 15 mm AP diameter on TVS. Time limit not stated (several weeks)	Maternal age, parity, menstrual age, gestational sac (yes or no), gestational sac diameter, bleeding before inclusion (days), pain before inclusion (days), previous miscarriage, previous termination of pregnancy Included in multivariable: Gestational sac on TVS (yes or no), course and amount of bleeding, abdominal pain (yes or no)	Only women with bleeding analyzed (*n* = 142): Results not reported	Only women with bleeding analyzed (*n* = 142): Increasing bleeding after inclusion (+)
Combined randomized and non-randomized (expectant vs. D&E)						
* N* = 188						
Memtsa 2017 [18]	64/83 (77%)	Missed miscarriage in first trimester (embryonic or anembryonic) Bleeding and/or pain	Urinary pregnancy test negative and bleeding ceased ≤ 7 days without D&E (time not clearly stated)	s-β-hCG, s-PAPP-A, s-hs-CRP, s-progesterone, Maternal age, menstrual age, ethnicity, BMI, smoker (yes or no), parity, pain (yes or no), amount of bleeding, gestational sac diameter, gestational sac volume	Maternal age (+),s- progesterone (−), s- PAPP-A (−)	Maternal age (+) and s-progesterone (−)
Prospective observational						
*N* = 83						
Casikar 2013 [15]	Training set: 74% (137/186); test set: 77% (97/126)	First-trimester miscarriages: Incomplete (65%) (heterogenous RPOC but no cut off in mm), embryonic (20%), anembryonic (15%); both bleeding and no bleeding	Complete resolution ≤ 2 weeks: cessation of vaginal bleeding and the absence of RPOC (no cut off) on TVS	Maternal age, menstrual age, previous vaginal delivery, previous cesarean section, previous miscarriage, previous termination of pregnancy, subtype of miscarriage, smoker (yes or no), amount of vaginal bleeding, endometrial thickness (only if incomplete miscarriage), RPOC volume (only if incomplete miscarriage), abdominal pain (yes or no)	Training set: maternal age (−), menstrual age (−), previous miscarriage (−), bleeding (+), symptoms (+), type of miscarriage, i.e., incomplete (+), embryonic and anembryonic(−)	Type of miscarriage, i.e., incomplete (+), embryonic and anembryonic (−), maternal age (−), vaginal bleeding (+)
Prospective observational						
* N* = 312; 186 (training set) + 126 (test set)						
Medical management						
Schreiber 2015 [32]	52% (49/95)	First-trimester pregnancy failure (embryonic, anembryonic); both bleeding and no bleeding	Complete expulsion of products of conception (≤ 30 mm AP diameter on TVS) ≤ 3 days	Maternal age, ethnicity, BMI, previous miscarriage, parity, miscarriage type, duration of bleeding, abdominal pain within last 24 h (yes or no), vaginal bleeding within last 24 h (yes or no), gestational sac mean diameter, s-Activin A, s-ADAM-12, s-human placental lactogen, s-glycodelin, s-progesterone, s-estradiol, s-β-hCG	Parity (−) Hispanic ethnicity (−) s-ADAM-12 (−)	Hispanic ethnicity (−), parity (−), s- β-hCG ≥ 4000 (+), s-ADAM-12 ≥ 2500 (+)
Retrospective sub-analysis of a randomized controlled multicenter trial. Misoprostol 800 µg per vaginam; single dose						
* N* = 95						
Creinin 2006 [26]	84% (410/485) (single or repeated dose)	Anembryonic (36%), embryonic (58%), incomplete (endometrial lining > 30 mm) and inevitable (gestational sac and open cervical os and vaginal bleeding) (6%)	Complete miscarriage (≤ 30 mm AP diameter on TVS) without D&E ≤ 30 days (D&E on day 8 if still RPOC)	Abdominal pain within last 24 h (yes or no), vaginal bleeding within last 24 h (yes or no), Rh-type, type of miscarriage, parity, fever/chills during last week (yes or no), status of cervical os (closed or open), blood in vagina, maternal age, race, menstrual age, number of previous pregnancies, planned pregnancy (yes or no), s-β-hCG, Hb, number of previous miscarriages, height, weight, social and cultural variables, and more	Abdominal pain within last 24 h (+), vaginal bleeding within last 24 h (+), Rh-negative blood type (+), type of pregnancy failure, i.e., incomplete/inevitable (+ +), embryonic (+), anembryonic (−), no people younger than 18 years in the household (+)	Single-dose success: Vaginal bleeding within last 24 h (+) and nulliparity (+), Overall success: Localized abdominal pain within last 24 h (+), vaginal bleeding within last 24 h (+), Rh-negative blood type (+), nulliparity (+)
Planned substudy of multicenter randomized trial. Misoprostol 800 µg per vaginam; single dose per vaginam; or, if needed (gestational sac or endometrial lining > 30 mm) a second dose after 3 days						
* N* = 485						
Odeh 2010 [31]	40% (32/81) (one or two doses)	Anembryonic gestation (16%), embryonic (84%) < 12 weeks according to ultrasound examination; probably no bleeding	Endometrial lining < 30 mm on TVS within 12–24 h	Maternal age, menstrual age, previous delivery, previous abortions, previous pregnancies, CRL, gestational sac volume, s-β-hCG	Previous deliveries (−), s-β-hCG (−)	Not performed
Retrospective cohort. Misoprostol 800 µg per vaginam						
* N* = 81						
Agostini 2005 [23]	65% (180/276)	Anembryonic (gestational sac < 75 mm) or embryonic(CRL < 50 mm); both bleeding or no bleeding	Complete expulsion of pregnancy products (endometrial thickness < 15 mm) within 24 h	Maternal age, menstrual age, previous pregnancies, parity, previous vaginal deliveries, previous spontaneous abortions, CRL, mean gestational sac diameter	Previous pregnancies (−), parity (−), previous vaginal deliveries (−)	Parity: para ≤ 1 (+), para ≥ 2 (−)
Prospective observational Misoprostol 800 µg per vaginam						
* N* = 276						
Lavecchia 2015 [28]	No D&E: 80% (155/199) No unplanned return to emergency department (URED): 69% (137/199)	Emergency department prescription for medical management and pregnancy arrest < 8 weeks according to ultrasound: missed abortion (33%), anembryonic gestation (28%), incomplete abortion (39%); no definitions of miscarriage types; at least some patients had bleeding	No D&E and no URED, observation time not stated	Maternal age, parity, previous cesarean delivery, previous abortions, type of miscarriage, menstrual age	Menstrual age ≤ 8 weeks (+), menstrual age > 8 weeks (−)	Menstrual age (−)
Retrospective cohort Different and not defined misoprostol regimes						
* N* = 199						
Lavecchia 2016 [29]	No D&E 80% (182/227) No unplanned return to the emergency department (URED): 70% (159/227)	Emergency department prescription for medical management and pregnancy arrest < 8 weeks according to ultrasound examination: missed abortion (38%), anembryonic (28%), incomplete (34%); no definitions of miscarriage types; at least some patients had bleeding	No D&E and no URED, observation time not stated The aim was to identify predictors of failure	Uterine cavity sonographic measurements, maternal age, gestational age, previous abortions, previous cesarean delivery, parity	Uterine cavity diameter (−) (for no D&E and no URED), uterine cavity volume (−) (for no URED), uterine cavity length (−) (for no URED)	Uterine cavity diameter > 15 mm was an independent risk factor for the need of D&E and URED
Secondary analysis of retrospective observational cohort Different not defined misoprostol regimes						
* N* = 227						
Jung In Kim 2017 [27]	94% (209/222)	Miscarriage (embryonic and anembryonic) ≤ 11 weeks; both bleeding and no bleeding	Complete expulsion of the conceptus (not defined) without D&E ≤ 24 h	Maternal age, menstrual age, parity, previous cesarean delivery, previous vaginal delivery, previous curettage, type of miscarriage, mean gestational sac diameter, CRL, uterine myoma (yes/no), uterine adenomyosis (yes/no), s-β-hCG	Previous vaginal delivery (no patient in the failure group had a previous vaginal delivery), s-β-hCG (−)	s-β-hCG > 40,000 was significantly associated with failed medical management ≤ 24 h
Retrospective cohort 1–3 doses of Misoprostol 800 µg per vaginam;						
* N* = 228						
Banerjee 2013 [24]	77% (40/52)	Embryonic or anembryonic < 12 weeks, no active bleeding	Complete miscarriage (not defined) ≤ 72 h	Maternal age, menstrual age, parity, s-progesterone	s-progesterone (+)	Mifepristone + misoprostol was less effective if s-progesterone was < 10 nmol/L
Prospective observational Mifepristone and misoprostol						
* N* = 53						

*AP* diameter, anterior–posterior diameter of intrauterine contents on transvaginal ultrasound *BMI* body mass index, *CRL* crown-rump length, *RPOC* retained products of conception, *TVS* transvaginal ultrasound, *D&E* dilatation and evacuation, *hCG* human chorionic gonadotropin, *Hb* hemoglobin, *PI* pulsatility index, *RI* resistance index, *PSV* peak systolic velocity, *PAPP-A* pregnancy associated plasma protein-A, *hs-CRP* high sensitivity C-reactive protein, *URED* unplanned return to emergency department

^a^The table is not based on a systematic literature search

^b^(+) the higher the value of the variable tested the higher the success rate of miscarriage management, alternatively if the variable was present the likelihood of success increased; (−) the higher the value of the variable tested the lower the success rate of miscarriage management, alternatively if the variable was present the likelihood of success decreased

Our results seem plausible from a pathophysiological perspective. Higher concentrations of s-β-hCG and s-progesterone probably reflect better functioning corpus luteum and trophoblast and are compatible with earlier stages of miscarriage explaining longer time to complete miscarriage. The relation between gestational age according to LMP, crown-rump-length and gestational sac diameter may reflect the time since the embryo died. The apoptotic process may be faster for the gestational sac than for the embryo. This could be an explanation for a smaller gestational sac being a predictor of treatment success. Long time between embryonic death and start of bleeding may also indicate resistance to expulsion. In some anembryonic miscarriages, the absence of an embryo might be explained by the embryo having been resorbed after having been dead for a long time.

The likely reason why we did not find any predictor of successful misoprostol treatment is that misoprostol is very effective in most patients. Results of other studies investigating predictors of successful misoprostol treatment are extremely variable (Table 8) [23, 24, 26–29, 31, 32] and no study is directly comparable to ours. The variable results are probably explained by differences in patient selection, definition of treatment success and predictor variables tested.

Some women prefer expectant management to medical intervention [17, 49] and knowledge about prognostic factors is important for being able to provide them with realistic expectations of treatment success. Our prediction models are helpful in this respect but need to be prospectively validated. A simple blood test predicting successful outcome without treatment would be clinically valuable and therefore a goal of future research. Serum-progesterone or serum-hCG results may not always be available at the time of patient counselling. Therefore, they may be less clinically useful as predictors than clinical and ultrasound information available bedside.
